# Targeting *Candida albicans* Pathogenicity: A Multifactorial Approach Using *Lippia graveolens* Essential Oil

**DOI:** 10.3390/ijms27010166

**Published:** 2025-12-23

**Authors:** Mario Rodriguez-Canales, Ana Bertha Hernandez-Hernandez, Uriel Nava-Solis, Marco Aurelio Rodriguez-Monroy, Maria Margarita Canales-Martinez

**Affiliations:** 1 Laboratorio de Farmacognosia, Unidad de Biotecnología y Prototipos, Facultad de Estudios Superiores Iztacala, Universidad Nacional Autónoma de México, Av. de los Barrios No. 1. Los Reyes Iztacala, Tlalnepantla de Baz 54090, Mexico; ana.b.hdez@iztacala.unam.mx (A.B.H.-H.); biol.navasolis@gmail.com (U.N.-S.); 2 Laboratorio de Investigación Biomédica en Productos Naturales, Carrera Medicina, Facultad de Estudios Superiores Iztacala, Universidad Nacional Autónoma de México, Av. de los Barrios No. 1. Los Reyes Iztacala, Tlalnepantla de Baz 54090, Mexico; dr.marcorodriguezmonroy@gmail.com

**Keywords:** *Candida albicans*, Mexican oregano, essential oil, antifungal, germ tube inhibition, biofilm suppression, virulence factors, membrane integrity, natural products

## Abstract

*Candida albicans* is a major opportunistic fungal pathogen whose increasing resistance to antifungal agents requires new alternative therapies. This study evaluated the antifungal potential of *Lippia graveolens* (Mexican oregano) essential oil, with particular emphasis on its effects on key *C. albicans* virulence factors. The chemical composition of the essential oil was determined by GC–MS, identifying carvacrol and thymol as abundant components. Antifungal activity was assessed via disk diffusion, broth microdilution, and time-kill kinetics against clinical and reference *Candida* strains. The essential oil showed potent fungicidal activity, with MIC and MFC values of 2 mg/mL and 3 mg/mL, respectively. In addition to demonstrating antifungal potency, this work focused on *C. albicans* virulence factors, revealing that *L. graveolens* essential oil significantly inhibited germ tube formation at 1 mg/mL and completely suppressed both germ tube and biofilm development at concentrations ≥ 2 mg/mL, along with dose-dependent disruption of fungal membrane integrity. These findings highlight the multifactorial mechanisms by which *L. graveolens* essential oil affects *C. albicans* pathogenicity. This study supports its potential as a natural antifungal agent and a valuable adjuvant in the treatment of resistant candidiasis.

## 1. Introduction

*Candida* species are commensal yeasts found asymptomatically on human skin and mucous membranes, such as the gastrointestinal and reproductive tracts, oral cavity, and even the blood of humans and several animals [1]. These yeasts can act as opportunistic pathogens in individuals with microbiota alterations or environmental changes, such as shifts in pH and nutritional content, particularly in immunosuppressed hosts [2]. This includes individuals with AIDS, those undergoing cancer treatment, those receiving immunosuppressive therapy for organ transplantation, and the increasing elderly population [3]. Among the *Candida* species, *C. albicans* is the most prevalent, being the most common nosocomial pathogen in hospitals and health care centers. It accounts for approximately 70% of invasive fungal infections worldwide, causing diseases ranging from superficial skin infections to severe disseminated diseases with a high mortality rate of up to 40% [4]. However, recent epidemiological studies have noted a gradual decline in the relative proportion of infections caused by *C. albicans*, primarily due to the increasing prevalence of infections by other *Candida* species such as *C. glabrata*, *C. parapsilosis*, and *C. auris*. Despite this shift, *C. albicans* continues to represent the main etiological agent of invasive candidiasis, maintaining high clinical relevance and public health importance [4].

The immunocompromised state of the host is not the sole factor promoting opportunistic *C. albicans* infections; the virulence factors of *C. albicans* also play significant roles in its pathogenesis and disease severity [2,5]. These mechanisms include molecules that mediate adhesion and invasion into host cells, the secretion of hydrolases, the yeast-to-hypha phenotypic transition, contact sensing, and biofilm formation [2]. With the increase in invasive medical procedures and the growing population of immunocompromised individuals, *C. albicans* infections are becoming more common [3].

Currently, there are limited antifungal agents available to treat candidiasis, including polyenes, fluoropyrimidines, echinocandins, and azoles [6], with the latter being the preferred and most frequently used drugs for treating *C. albicans* infections [7]. However, the development of resistance among *Candida* species, particularly concerning azoles, has been documented extensively [4,6,8,9]. Consequently, controlling *C. albicans* infections presents a significant challenge in modern medicine, highlighting the need to continue researching and developing new antifungal alternatives to address this problem.

Owing to their broad therapeutic effects and low toxicity, natural products and their chemical compounds have been proposed as candidates for the future development of new medicines [10,11]. Essential oils are complex mixtures of volatile organic compounds characterized by their strong fragrance. These compounds are produced by various plant species as secondary metabolites and can be extracted from leaves, flowers, fruits, seeds, roots, buds, stems, and wood [12]. Their chemical composition is influenced by many factors, including plant species, geographic location, ecotype, soil physical-chemical properties, nutrition, and stress during growth, among others.

Essential oils rich in phenols are known to exhibit activity against several bacterial and fungal pathogens, in addition to possessing antioxidant, anticancer, immunomodulatory, analgesic, and anti-inflammatory activities [13]. Owing to these properties, essential oils are natural products that can be tested against pathogens of human interest, such as *C. albicans*, in the search for new therapeutic options against fungal infections.

Accordingly, many studies have demonstrated that some essential oils have antifungal activity, including their ability to disrupt fungal cell membranes, inhibit the synthesis of essential cellular components, and interfere with fungal metabolism [14,15,16,17,18].

Mexican oregano (*Lippia graveolens* Kunth) essential oil has been widely studied due to its diverse biomedical properties. For example, its anti-inflammatory and antioxidant activities have been demonstrated, showing its ability to reduce the production of reactive oxygen species (ROS) and nitric oxide (NO) [19,20]. Additionally, essential oils have demonstrated notable antimicrobial properties against a range of pathogens, including multidrug-resistant strains of *Enterococcus faecalis* and *Staphylococcus aureus* [21]. The essential oil also possesses antimutagenic properties, as evidenced by its ability to inhibit mutagenicity induced by chemicals such as 4-nitro-o-phenylenadiamine and sodium azide; this activity is attributed to the high phenolic content in the oil [22]. Moreover, the oil has shown cytotoxic effects against the U-251 glioblastoma cancer cell line and the SK-LU-1 lung cancer cell line and could be harnessed for cancer treatment [20].

Nevertheless, only a few studies have addressed the antifungal properties of the essential oil of Mexican oregano. Previous research has highlighted the general antifungal properties of Mexican oregano essential oil [23], and our work aims to provide a more detailed understanding of its effects on several *C. albicans*-specific virulence factors, such as yeast-to-hypha transition, germ tube formation, biofilm formation and the integrity of the cell membrane and cell wall.

The anti-*C. albicans* properties and mechanisms of action of *L. graveolens* essential oil remain underexplored, in contrast with those reported for other essential oils of plants that are also known as “oregano”, such as *Origanum vulgare* (European oregano) essential oil, where its antifungal effects on *C. albicans* virulence factors have been widely investigated. In these works, the essential oil has been shown to be capable of inhibiting biofilm formation, hyphal growth, and adhesion to host cells, thereby reducing the virulence of *C. albicans* [24,25]; this effect is likely due to the combined action of compounds such as carvacrol and thymol, which are identified as key active components responsible for these effects [24,25]. The lack of detailed studies on the specific impacts of *L. graveolens* essential oil on the virulence factors of *C. albicans* emphasizes the importance of fully understanding its potential as a therapeutic agent against *C. albicans* infections.

Given the increasing incidence of antifungal resistance and the limited number of effective antifungal agents available [26], the exploration of natural products such as Mexican oregano essential oil as alternative treatments is highly important. Further research into the antifungal properties and mechanisms of action of these natural products will contribute to the development of new, effective treatments for *C. albicans* infections, addressing a critical need in modern medicine. Importantly, it will also reinforce and validate the use of this natural product to treat common and recurrent skin and nail infections caused by fungus, as part of the practices of traditional Mexican medicine and as an alternative/adjuvant to conventional treatments, which for a large sector of the population may seem inaccessible owing to their costs and availability.

Therefore, this study aimed to investigate the antifungal activity of *L. graveolens* essential oil against *C. albicans*, with a particular focus on its effects on key virulence factors, including germ tube formation, biofilm development, and membrane integrity. Our findings reveal that the essential oil has multifactorial antifungal effects, highlighting its potential as a natural therapeutic agent or adjuvant treatment for resistant *Candida* infections.

## 2. Results

### 2.1. L. graveolens Essential Oil Chemical Composition

The chemical composition of *L. graveolens* essential oil was analyzed using gas chromatography-mass spectrometry (GC-MS), and 27 compounds were identified (Table 1). These include some phenolic monoterpenoids, terpene hydrocarbons, and oxygenated derivatives, which are known for their biological properties, particularly their antifungal effects. Among the most relevant compounds identified are carvacrol, thymol, eucalyptol, o-cymene and terpinene, all of which are recognized for their antifungal properties.

The presence of carvacrol and thymol, both of which are considered hallmark constituents of *L. graveolens* and other species, such as *O. vulgare*, is particularly significant; both compounds are associated with strong antifungal effects and play key roles in disrupting fungal cell membranes and inhibiting biofilm formation. Additionally, eucalyptol, an oxygenated monoterpene, complements these effects by interfering with fungal morphogenesis and reducing biofilm development. Furthermore, o-cymene and γ-terpinene, although not directly antifungal, enhance the activity of carvacrol and thymol, contributing to the overall effectiveness of the essential oil.

The presence of these bioactive compounds highlights the complex and synergistic interactions within the essential oil that most likely contribute to its observed antifungal activity. Importantly, this mixture of chemical compounds present in *L. graveolens* essential oil reduces the likelihood of resistance development by fungi because of its multiple and simultaneous targeting of important pathways, making it difficult for yeast cells to adapt to or develop resistance mechanisms against all these compounds.

### 2.2. L. graveolens Essential Oil Inhibits the Growth of Different Candida Strains

In the disk diffusion assay method, all the *Candida* pathogen strains tested showed complete inhibition of fungal growth.

Owing to the total growth inhibition, the inhibition zone was determined by measuring the distance from the application point of the impregnated disk to the edge of the Petri dish (considering this as the radius), yielding an inhibition zone diameter of 30 mm. This finding indicates that the essential oil effectively prevents fungal proliferation across the entire culture surface. This biological effect was observed in both the catalog strains and clinical isolates from resistant cases (Table 2); these results are especially significant in recurrent fungal infections, where resistant strains and biofilm-associated pathogens often play a dominant role.

The experiments were conducted in triplicate under controlled and reproducible conditions, where nystatin was used as a positive control and significantly inhibited all *Candida* pathogen strains, but growth was visible; Petri dishes with *Candida* strains and without treatments were used as the negative control. Traditional fungal growth was observed on these control plates.

The antifungal activity of *L. graveolens* essential oil was further quantified through broth microdilution assays to determine its Minimum Inhibitory Concentration (MIC) and Minimum Fungicidal Concentration (MFC) against all *Candida* pathogen strains tested, including clinical isolates and catalog strains (Table 2). The MIC required to inhibit growth in all strains tested was consistently 2 mg/mL, whereas the MFC needed to achieve complete fungal eradication was 3 mg/mL.

### 2.3. Time-Kill Kinetics of L. graveolens Essential Oil Against C. albicans

**Figure 1:**
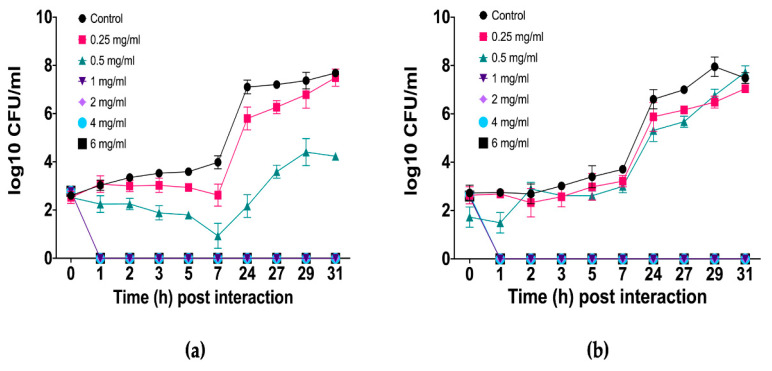
Time-kill kinetics of *L. graveolens* essential oil against (**a**) *C. albicans* (resistant clinical isolate) and (**b**) *C. albicans* ATCC 10231/CDBB-L-1003. The effects of various concentrations of *L. graveolens* essential oil (0.25, 0.5, 1, 2, 4, and 6 mg/mL) on fungal growth were tested, and fungal growth was monitored over 31 h. The fungicidal effect was observed at 1 mg/mL or higher; the fungistatic effect was observed at 0.5 mg/mL.

Time-kill kinetics assays were conducted exclusively on *C. albicans* strains, given that the fungicidal effect of *L. graveolens* essential oil had already been demonstrated in all tested *Candida* pathogen strains. This focus was also justified by the clinical relevance of *C. albicans* as the most pathogenic and prevalent species, as it is frequently associated with both superficial and systemic infections. The results revealed a dose-dependent antifungal effect, with complete elimination of fungal cells observed at 1 mg/mL (1/2 MIC) or higher concentrations; the fungal population decreased to undetectable levels within the first hour and remained suppressed throughout the 31 h assay, indicating a sustained fungicidal effect (Figure 1).

At 0.5 mg/mL, the essential oil had a fungistatic effect on *C. albicans* resistant clinical isolate (Figure 1a), as fungal growth was inhibited during the first 7 h, after which a gradual recovery of the fungal population was observed. However, even at the end of the assay, the population remained significantly lower (log_10_ 3 and 4) than that of the untreated control (log_10_ 8). In contrast, the essential oil was less effective against *C. albicans* ATCC 10231/CDBB-L-1003 (Figure 1b), whose growth pattern resembled that of the untreated control group. Nevertheless, at several time points, fungistatic activity was evident and statistically confirmed. At 0.25 mg/mL, in both strains the fungal population presented a growth pattern very similar to that of the untreated control, with only a slight delay in growth.

### 2.4. Inhibition of C. albicans Germinative Tube Formation by L. graveolens Essential Oil

**Figure 2:**
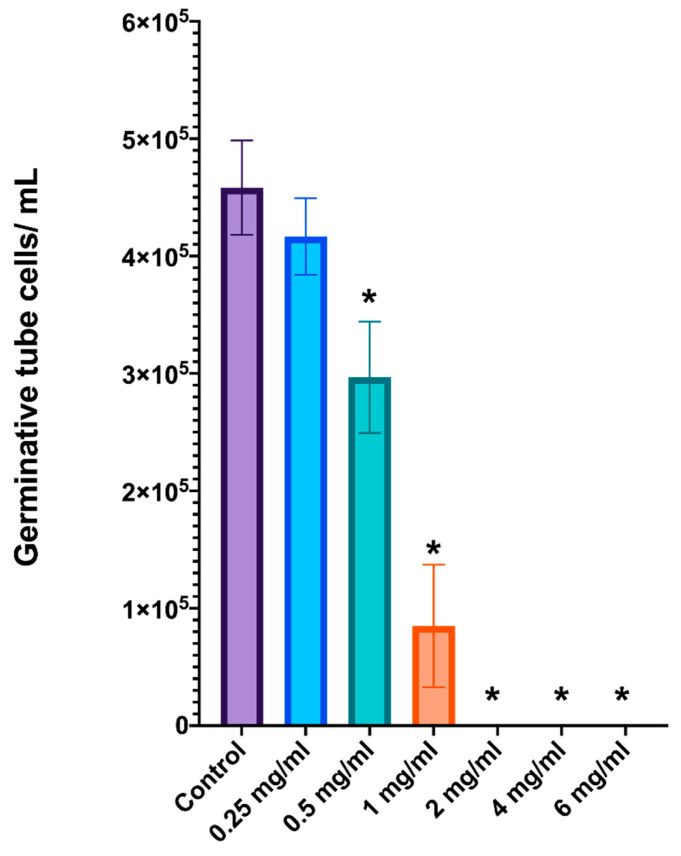
Inhibition of germinative tube formation in *C. albicans* ATCC 10231/CDBB-L-1003 by *L. graveolens* essential oil. The number of germinative tube cells/mL was quantified using a Neubauer chamber after treatment with different concentrations of *L. graveolens* essential oil (0.25, 0.5, 1, 2, 4, and 6 mg/mL). The control group presented the greatest number of germinative tube cells (5 × 10^5^ cells/mL), whereas a concentration of 1 mg/mL significantly reduced germ tube formation (*p* < 0.05). Complete inhibition was observed at concentrations ≥ 2 mg/mL. Statistical analysis was performed using one-way ANOVA (*p* < 0.05). * indicates a significant difference compared with the control group. The data are presented as the means ± standard deviations of three independent experiments.

The effects of *L. graveolens* essential oil on germinative tube formation in *C. albicans* were evaluated with cell counting in a Neubauer chamber. The results demonstrated a clear dose-dependent inhibition of germinative tube formation (Figure 2). The untreated control group presented the greatest number of cells with germinative tubes, with approximately 5 × 10^5^ cells/mL, which was consistent with typical germ tube formation under inducing conditions. At low concentrations of 0.25 and 0.5 mg/mL, the essential oil clearly reduced the ability of *C. albicans* to form a germinative tube structure but did not kill the yeast. However, a statistically significant decrease (one-way ANOVA; *p* < 0.05) was observed at 1 mg/mL, where the number of germinative tube cells was reduced to approximately 8.5 × 10^3^ cells/mL. Furthermore, at concentrations of 2 mg/mL or greater, germinative tube formation was completely inhibited, with no detectable germinative tube cells in the Neubauer chamber and a significant reduction in live yeast cells. These results indicate that essential oil at low concentrations can effectively block germ tube formation and, at concentrations of 2 mg/mL or greater, completely inhibit the formation of this critical virulence factor in *C. albicans*.

This dose-dependent inhibition of germinative tube formation underscores the potential of *L. graveolens* essential oil to suppress the pathogenicity of *C. albicans* by targeting its ability to transition to the hyphal form.

### 2.5. Effect of L. graveolens Essential Oil on the Cell Membrane Integrity of C. albicans

**Figure 3:**
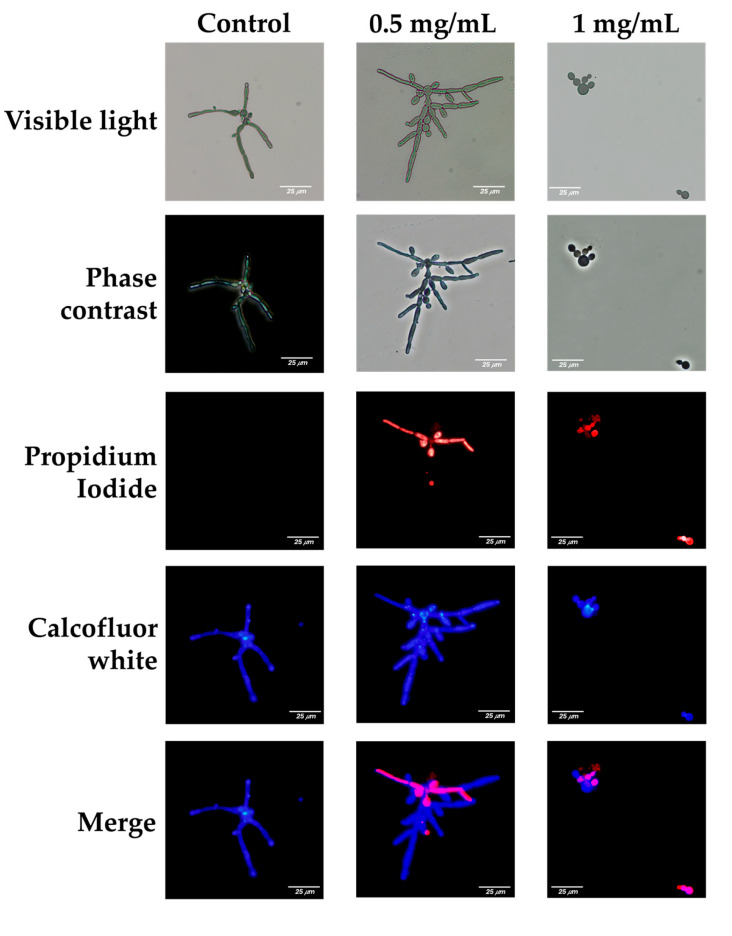
Disruptive effects of *L. graveolens* essential oil on the integrity of the cell membrane of *C. albicans* ATCC 10231/CDBB-L-1003. Microscopic analysis of *C. albicans* treated with 0.5 or 1 mg/mL *L. graveolens* essential oil compared with the untreated control. The figures shown are representative microphotographs of each experimental group. Scale bar = 25 µm (all panels).

The impact of *L. graveolens* essential oil on the integrity of the cell membrane of *C. albicans* was evaluated using propidium iodide (PI) and calcofluor-white (CFW) staining, observed under visible light, phase contrast, and fluorescence microscopy. Red fluorescence (PI) indicates membrane damage, as PI is impermeable to cells with intact membranes. Blue fluorescence (CFW) highlights the chitin and cellulose in fungal cell walls. Representative images are shown for untreated control cells and cells treated with 0.5 mg/mL or 1 mg/mL essential oil (Figure 3).

In the control group, germinative tube formation was observed under visible light and phase contrast microscopy, which revealed elongated structures and hyphal cells typical of *C. albicans* cultured under favorable growth conditions. Fluorescence microscopy revealed intense blue fluorescence (CFW), reflecting intact cell wall structures, and minimal or no red fluorescence (PI), indicating no membrane damage.

At a concentration of 0.5 mg/mL, germinative tube formation persisted, with elongated hyphal structures characteristic of *C. albicans*. However, fluorescence microscopy revealed significant membrane damage in a subset of the population, as evidenced by the red fluorescence emitted by propidium iodide. The presence of this red fluorescence in germinative tube cells suggests that, despite their ability to form hyphae, their membrane integrity is partially disrupted at 0.5 mg/mL of the essential oil (Figure 3).

Such compromised cells are likely less capable of withstanding additional challenges, including host immune responses and/or the action of antifungal treatments. The immune system, particularly phagocytic cells such as macrophages and neutrophils, may exploit this weakened state to address infection more effectively. Additionally, disruption of the fungal cell membrane could facilitate the uptake of antifungal drugs or synergize with other treatments, increasing their efficacy by reducing the ability of fungal cells to mount resistance mechanisms.

At 1 mg/mL of *L. graveolens* essential oil, the antifungal effects were markedly pronounced, with significant impacts on the viability and morphology of *C. albicans*. Microscopy analysis revealed an almost complete absence of fungal cells within the preparations, indicating substantial cell death. It was challenging to identify viable yeast cells under these conditions, suggesting that the essential oil at this concentration severely impaired fungal survival. Red fluorescence from propidium iodide staining was absent or undetectable, which may reflect the near-complete elimination of fungal cells rather than intact membrane integrity, as viable cells were scarce. Compared with that at lower concentrations, the amount of calcofluor-white staining, while still present, was significantly lower.

Importantly, the few cells that were observed were all in yeast form, and germinative tube formation was completely absent.

### 2.6. Inhibition of C. albicans Biofilm Formation by L. graveolens Essential Oil

**Figure 4:**
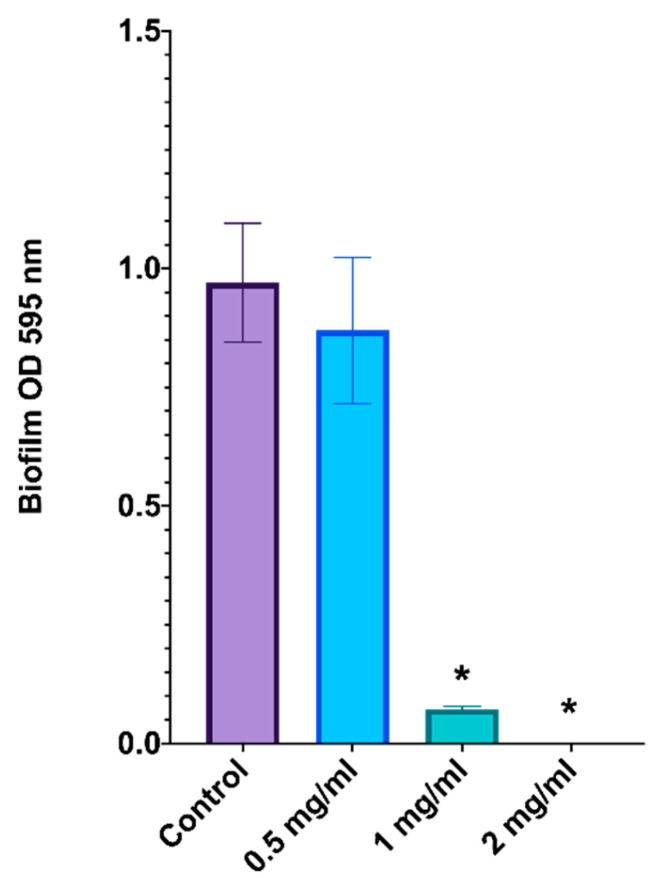
Inhibition of *C. albicans* ATCC 10231/CDBB-L-1003 biofilm formation by *L. graveolens* essential oil. The optical density (OD) of the biofilm biomass was measured at 595 nm after it was stained with crystal violet. Statistical analysis was performed using one-way ANOVA (*p* < 0.05). * represents significant differences compared with the control group. The data represent the means ± standard deviations of three independent experiments.

The effect of *L. graveolens* essential oil on *C. albicans* biofilm formation was evaluated by determining the optical density (OD) at 595 nm after the biofilms were stained with crystal violet (Figure 4). These results indicate a dose-dependent inhibition of biofilm formation.

In the untreated control group, robust biofilm formation was observed, with an OD of approximately 1.0, indicating optimal conditions for biofilm development. At 0.5 mg/mL of the essential oil, there was a slight reduction in biofilm formation, but the difference was not statistically significant compared with that of the control group.

At a dose of 1 mg/mL, biofilm formation was significantly inhibited, with the OD reduced to less than 0.1 after crystal violet staining, corresponding to more than 90% inhibition relative to the control (*p* < 0.05). This result highlights the substantial disruption of fungal biofilm biomass at this concentration. At 2 mg/mL, *L. graveolens* essential oil completely suppressed biofilm formation, with an OD close to zero, suggesting complete inhibition (*p* < 0.05).

## 3. Discussion

The findings of this study strongly support the antifungal potential of *L. graveolens* essential oil against different *Candida* strains, highlighting its effect against *C. albicans*, including both reference strains and clinical isolates resistant to conventional antifungal agents. *L. graveolens* essential oil consistently inhibited fungal growth, with inhibition halos of 30 mm in disk diffusion assays. These results align with those reported in other studies, where *Lippia* spp. essential oil has shown inhibition halos ranging from 10 mm to 14 mm, depending on the experimental conditions and strain susceptibility. For example, inhibition halos of approximately 13 mm against *C. albicans* clinical isolates were reported when paper disks were impregnated with 15 µL of essential oil [27]. The variations observed may be attributable to differences in the chemical composition of the essential oil or to methodological factors such as the amount of oil used on the paper disks or the growth medium used.

The inhibition halos observed in this study are also slightly greater than those reported for other related oregano species, such as *O. vulgare* (European oregano). For example, inhibition halos of 15–18 mm have been reported for *O. vulgare* essential oil under similar experimental conditions [28]. These differences in antifungal activity between these species can likely be attributed to their distinct chemical profiles. Nevertheless, although both oils contain carvacrol and thymol as major active components, *L. graveolens* typically has a relatively high relative content of carvacrol, which has been linked to relatively strong fungal membrane-disrupting properties [29]. Furthermore, the presence of additional compounds, such as *o*-cymene and γ-terpinene, may increase the bioavailability and synergistic effects of phenolic monoterpenoids in *L. graveolens* because these compounds are key biosynthetic precursors of both carvacrol and thymol [30,31]. Additionally, the lower polarities of *o*-cymene and γ-terpinene than carvacrol and thymol enable them to penetrate lipid-rich fungal membranes more effectively [32,33]. This dual role as a biosynthetic intermediate and synergistic facilitator may enhance the antifungal efficacy of the essential oil by potentially improving the availability of the active compounds at their target sites.

The fungicidal properties of *L. graveolens* essential oil were further confirmed by its MIC and MFC values, which were determined to be 2 mg/mL and 3 mg/mL, respectively. These findings underscore the robustness of its antifungal activity across different studies and reinforce the therapeutic potential of this essential oil. The synergistic activity between major and minor constituents, such as carvacrol, thymol, *o*-cymene and γ-terpinene, plays a crucial role in enhancing the overall activity of the essential oil.

Regarding this, several essential oils evaluated against *Candida* spp. have demonstrated wide variability in antifungal potency when expressed as minimum inhibitory concentrations (MICs). Reported MIC values for whole essential oils typically span a broad range, from µg/mL to several mg/mL. For instance, antifungal MIC values for different essential oils against *Candida* species have been reported to range from 0.0008 to 0.8 mg/mL, with marked differences among oils and fungal species [16].

Similarly, other studies have reported MIC values for essential oils against *Candida* spp., ranging from below 1 mg/mL to 5 mg/mL or higher, particularly for complex multicomponent oils rather than isolated compounds [34,35]. In this context, the MIC value of 2 mg/mL observed for *L. graveolens* essential oil falls within this reported range for several essential oils and is consistent with values described for other chemically complex plant-derived oils.

The growth kinetics analysis of *C. albicans* in the presence of different concentrations of *L. graveolens* essential oil revealed a dose-dependent antifungal effect. At concentrations of 1 mg/mL and above, the essential oil had a fungicidal effect, as evidenced by the suppression of fungal growth beginning at 1 h post interaction and throughout the entire experimental period. In contrast, at 0.5 mg/mL, a fungistatic effect was observed, where initial growth was inhibited, whereas after a period, fungal proliferation resumed but at a minor scale compared with growth in the control group. At the lowest concentration tested (0.25 mg/mL), the growth curve closely resembled that of the untreated control, indicating minimal inhibitory activity.

The fungicidal activity observed at concentrations of 1 mg/mL and higher is consistent with previous reports on carvacrol and thymol-rich essential oils, where these phenolic monoterpenoids showed the ability to disrupt fungal membrane integrity, leading to rapid cell death [36,37,38].

At a concentration of 0.5 mg/mL, where a fungistatic effect was observed, it is likely that the essential oil components were present at sublethal concentrations, sufficient to slow fungal metabolism but not to cause irreversible damage. This phenomenon may be due to the low concentrations of carvacrol and thymol, which can induce stress responses in fungal cells [39], triggering adaptive mechanisms that allow recovery once the antifungal pressure is reduced [40]. The resumption of growth after initial inhibition suggests that *C. albicans* may be capable of compensatory responses, potentially mediated by the upregulation of stress response genes or modifications in membrane composition [40].

The inability of low concentrations to inhibit fungal growth reinforces the importance of reaching threshold levels for essential oil efficacy, particularly in clinical applications and/or traditional medicine contexts, where suboptimal dosing may not contribute to therapeutic outcomes.

The inhibition of germ tube formation in *C. albicans* by *L. graveolens* essential oil revealed a consistent concentration-dependent effect, with a transition from morphogenesis inhibition at 1 mg/mL to complete fungicidal action at 2 mg/mL and above. At 1 mg/mL, a significant reduction in hyphal formation was observed, indicating that the essential oil interfered with the morphogenetic switch necessary for *C. albicans* virulence. However, at 2 mg/mL and higher, drastic reductions in viable cells were evident, with the few remaining fungal cells appearing in yeast form rather than those with germinative tube structures or hyphae, suggesting a lethal effect at these concentrations.

*C. albicans* has a remarkable ability to switch between yeast and filamentous forms, a process crucial for its pathogenicity. The formation of germ tubes and hyphae facilitates adhesion to host tissues, tissue penetration, and biofilm development, which are key factors that increase fungal survival and virulence [41]. This morphogenetic transition is regulated by complex signaling networks that respond to external stimuli such as temperature, pH changes, and the presence of serum, with critical roles played by pathways such as Ras1, cAMP, PKA and MAPK, which coordinate growth from adaptation and virulence expression [42].

The inhibition at 1 mg/mL may be attributed to the action of carvacrol and thymol, owing to their known capacity to disrupt fungal membrane integrity and interfere with cellular signaling. Studies have shown that these compounds downregulate hypha-specific genes such as *ECE1*, *HWP1*, and *ALS3*, which are crucial for filamentation and host tissue adhesion [43,44]. Moreover, by altering membrane fluidity and impairing ergosterol biosynthesis, carvacrol compromises essential signaling cascades necessary for fungal morphogenesis and virulence [44].

At concentrations of 2 mg/mL and higher, the fungicidal effect became evident, with the complete absence of viable hyphal cells. The few remaining viable cells were exclusively in yeast form, indicating that the essential oil not only inhibited the transition to the hyphal state but also had a lethal effect on the fungal population.

The complete inhibition of germ tube formation at lethal concentrations suggests that *L. graveolens* essential oil not only disrupts fungal growth but also impairs its ability to establish infection by targeting key virulence factors, as the yeast-to-hypha transition is crucial for biofilm formation and tissue invasion [42].

Biofilm formation is a complex, multistep process in which the yeast-to-hyphal transition plays a crucial role, facilitating adherence to surfaces and the development of structured communities with increased resistance to antifungal treatments [45,46].

At 1 mg/mL, biofilm formation was significantly reduced, suggesting that although some fungal cells remained viable, their ability to form a structured, protective matrix was impaired. These results are consistent with previous studies indicating that carvacrol and thymol interfere with the expression of key biofilm-associated genes, such as *HWP1*, *ALS3*, and *ECE1*, which are essential for adhesion, hyphal development, and extracellular matrix production [47,48]. Because hyphal growth is a critical step in biofilm maturation, the inhibition of germ tube formation likely contributed to the reduced biofilm mass observed at this concentration.

At 2 mg/mL or higher, biofilm formation was nearly abolished, with only a minimal number of yeast-form cells remaining attached to the substrate.

The relationship between biofilm inhibition and germ tube suppression underscores the importance of targeting fungal morphogenesis in antifungal strategies. Biofilm-associated infections are notoriously difficult to treat because of their increased resistance to conventional antifungal drugs and immune system defenses. By preventing hyphal formation and biofilm maturation simultaneously, *L. graveolens* essential oil represents a promising alternative for the management of *C. albicans* infections, particularly in biofilm-associated conditions such as catheter-related infections, denture stomatitis, and chronic mucosal candidiasis.

The effect of *L. graveolens* essential oil on *C. albicans* cell membrane integrity provides further insight into its antifungal mechanism of action and reinforces its ability to disrupt key fungal structures necessary for pathogenesis. Fluorescence microscopy images revealed that at 0.5 mg/mL, although fungal cells were still capable of forming pseudo hyphae and germ tubes, their membrane integrity was significantly compromised, as evidenced by the intracellular accumulation of propidium iodide (PI). PI is a membrane-impermeable dye that penetrates only cells with damaged plasma membranes, indicating that even at concentrations where fungal growth and morphogenesis are not entirely inhibited, *L. graveolens* essential oil has disruptive effects on fungal cell structure. This result aligns with previous findings on carvacrol and thymol, where sublethal concentrations induce membrane permeabilization without immediately causing cell death [49].

At 1 mg/mL, no germ tube formation was observed, and very few cells remained viable, all of which were in yeast form rather than in the filamentous state. These findings further support the idea that *L. graveolens* essential oil interferes with the yeast-to-hypha transition while simultaneously exerting fungicidal effects at relatively high concentrations. The drastic reduction in viable cells at this concentration is consistent with previous reports demonstrating that carvacrol and thymol target fungal membrane lipids, leading to increased permeability, leakage of essential intracellular components, and eventual cell death [49].

The combined effects of membrane permeabilization, inhibition of germ tube formation, and biofilm suppression highlight the multifaceted antifungal action of *L. graveolens* essential oil. Unlike conventional antifungal agents that typically target a single fungal component, such as ergosterol biosynthesis or cell wall synthesis, *L. graveolens* essential oil disrupts multiple fungal structures and pathways simultaneously. This broad-spectrum mechanism not only enhances its efficacy but also reduces the likelihood of resistance development, making it a promising antifungal agent candidate, particularly against resistant *C. albicans* strains [50,51].

Although the antimicrobial and antifungal properties of *L. graveolens* essential oil have been previously studied, its specific effects on critical virulence factors of *C. albicans* have not been thoroughly explored. This study provides new insights into the direct impact of *L. graveolens* essential oil on fundamental virulence factors of *C. albicans*, including germ tube formation, biofilm development, and membrane integrity, all of which are crucial for fungal adhesion, invasion, and persistence in host tissues.

As described previously, minor components of essential oils may not exhibit potent antimicrobial activity individually but can significantly potentiate the effects of major compounds through complementary mechanisms of action. According to the mechanistic framework described by Radulovic et al. [52], plant secondary metabolites frequently exert antimicrobial effects by targeting multiple cellular structures and processes, with the cytoplasmic membrane representing the primary site of action. In this context, *L. graveolens* essential oil, which features carvacrol and thymol as its major active components, is supported by monoterpene hydrocarbons such as p-cymene, γ-terpinene, α-terpinene, terpinolene and carenes. plant secondary metabolites frequently exert antimicrobial effects by targeting multiple cellular structures and processes, with the cytoplasmic membrane representing the primary site of action. In this context, *L. graveolens* essential oil, which features carvacrol and thymol as its primary active components, is supported by monoterpene hydrocarbons such as p-cymene, γ-terpinene, α-terpinene, terpinolene, and carenes. These compounds, although exhibiting limited antifungal activity individually, have been shown to modulate membrane fluidity and permeability, thereby facilitating the penetration and intracellular activity of more potent compounds, such as carvacrol and thymol [52]. Additionally, oxygenated monoterpenes detected in the oil, including linalool, terpineol, borneol, camphor, and eucalyptol, are reported to directly interact with the lipid bilayer, leading to membrane destabilization, leakage of intracellular contents, and disruption of cellular homeostasis. Therefore, the antifungal activity observed for L. graveolens essential oil is best interpreted as the result of a multifactorial, synergistic action of both major and minor constituents, acting through combined membrane-targeting and intracellular effects, rather than a single dominant compound.

Given the increasing prevalence of antifungal resistance, the ability of *L. graveolens* essential oil to simultaneously interfere with multiple fungal pathways presents a significant therapeutic advantage. Future research may explore its potential applications in clinical settings, including combination therapies with conventional antifungal drugs to increase its efficacy and mitigate resistance. Additionally, other in vivo studies are necessary to confirm the safety and effectiveness of *L. graveolens* essential oil in treating *C. albicans* infections. Overall, *L. graveolens* essential oil represents a promising natural antifungal agent with broad-spectrum activity and potential applications in fungal infection management.

## 4. Materials and Methods

### 4.1. Extraction of L. graveolens Essential Oil

#### 4.1.1. Collection of Plant Material

*L. graveolens* plant material was collected from Zapotitlán de las Salinas, between coordinates 18°07′18″ N and 97°19′24″–97°30′06″ W, at an altitude of 1200–2500 m above sea level. The collection took place in January 2020. Voucher specimens were deposited in the herbarium of the Universidad Nacional Autónoma de México (UNAM) under voucher number MCM8. The specimens were collected in the field with permission from the “Secretaría de Medio Ambiente y Recursos Naturales” (SGPA/DGVS/1266).

#### 4.1.2. Extraction of Essential Oil

*L. graveolens* branches were cut into 1–2 cm fragments using pruning shears. The essential oil was extracted using the hydrodistillation method [53]. In this process, 400 g of fresh plant material was placed in 500 mL of distilled water in a 1000 mL round-bottom flask equipped with a heating mantle (SEV-Prendo, MC301-9, Mexico City, Mexico) connected to a double-pass condenser designed by the Laboratory of Pharmacognosy at FES Iztacala, UNAM. The condenser was attached to a cold-water circulator set at a controlled temperature. Distillation proceeded for 40 min, and this process was repeated for all the plant materials. The essential oil was separated from the aqueous phase by density differences and stored in amber glass vials at −18 °C.

### 4.2. Chemical Analysis of L. graveolens Essential Oil by GC-MS

The essential oil of *L. graveolens* was analyzed using gas chromatography-mass spectrometry (GC-MS). The analysis was performed on an Agilent Technologies 6850 gas chromatograph coupled with a 5975C mass spectrometer (Agilent Technologies, Santa Clara, CA, USA) equipped with an HP-5MS column (30 m × 0.25 mm, 0.25 µm film thickness; Agilent Technologies). For the GC-MS analysis, 1 µL of the essential oil was injected in split mode. The initial oven temperature was set at 70 °C and held for 2 min, followed by a temperature ramp of 20 °C per minute to 230 °C; the temperature then increased at 8 °C per minute to 280 °C and held for 1 min. Helium was used as the carrier gas, with a constant flow rate maintained throughout the analysis. The ionization source temperature was set at 230 °C, and the mass spectrometer scanned a range of 35–750 *m*/*z* using electron impact ionization at 70 eV. The total analysis time was 17.25 min. The components of the essential oil were identified by comparing their mass spectra with the NIST version 8.0 library database (National Institute of Standards and Technology, Gaithersburg, MD, USA) and using retention indices for confirmation.

### 4.3. Antifungal Activity of L. graveolens Essential Oil

The antifungal activity of the *L. graveolens* essential oil was evaluated using the disk diffusion assay method (M44-A) following the guidance of the CLSI [54,55]. The assay was performed using both clinical and reference strains of *Candida* spp., as described below:

- *C. albicans* (clinical isolate): Donated by the Laboratory of Clinical Analysis, FES-CUSI, UNAM Iztacala.
- *C. albicans* ATCC 10231/CDBB-L-1003: Reference strain obtained from the CDBB strain collection (CINVESTAV, IPN, Mexico).
- *C. glabrata* (clinical isolate): Donated by the Laboratory of Clinical Analysis, FES-CUSI, UNAM Iztacala.
- *C. glabrata* CDBB-L-1536: Reference strain obtained from the CDBB strain collection (CINVESTAV, IPN, Mexico).
- *C. tropicalis* (clinical isolate): Donated by Hospital Los Angeles.
- *C. tropicalis* CDBB-L-1098: Reference strain obtained from the CDBB strain collection (CINVESTAV, IPN, Mexico).

All clinical isolates were confirmed using Candida Chromogenic Agar (CONDA, Cat. No. 1382.11), a chromogenic medium that enables presumptive identification of *Candida* species based on colony color and morphology.

The yeasts were grown for 48 h at 37 °C in 10 mL of RPMI-1640 medium (Sigma-Aldrich, St. Louis, MO, USA, Cat. No. R8758) supplemented with 2% glucose. Cultures were adjusted to 1.0 × 10^6^ CFU/mL by counting the yeasts in a Neubauer chamber and making the necessary dilutions with RPMI-1640 liquid medium. The yeast suspensions were cultured on Mueller-Hinton agar (2% glucose and 0.5 μg/mL methylene blue dye). Five-millimeter-diameter sterile paper disks were impregnated with 3 µL of essential oil, placed on the surface of agar plates previously inoculated and incubated at 37 °C for 48 h to ensure adequate growth.

After the incubation period, the antifungal activity was determined by measuring the diameter of the inhibition zones around the disks, including the diameter of the disk itself.

Nystatin disks were used as positive controls. The experiment was performed in triplicate to ensure the reproducibility and reliability of the results.

### 4.4. Broth Microdilution Assay

The antifungal activity of *L. graveolens* essential oil was further evaluated in a broth microdilution assay (M27-A3) [56]. The essential oil of *L. graveolens* was first dissolved in dimethyl sulfoxide (DMSO) before being added to the RPMI-1640 broth medium, at a final concentration of 0.2% (*v*/*v*). A DMSO tolerance curve previously performed demonstrated that concentrations up to 10% (*v*/*v*) did not affect the growth of any *Candida* yeast strains, confirming that the concentration used in this study was safe for antifungal testing. Nevertheless, all experiments included a DMSO control (yeast + DMSO) to corroborate the safety and reliability of the assay. Cultures of *Candida* strains were grown for 48 h at 37 °C in 10 mL of RPMI-1640 liquid medium (Sigma-Aldrich, Cat. No. R8758) supplemented with 2% glucose. The cultures were then adjusted to an initial concentration of 1 × 10^3^ yeast cells/mL by counting the cells in a Neubauer chamber and making the necessary dilutions with RPMI-1640 medium.

The assay was conducted in 96-well microtiter plates. Two-fold serial dilutions of the essential oil were first prepared directly in the wells, each containing 100 µL of the oil dissolved in RPMI-1640 medium. Subsequently, 100 µL of the *Candida* strain cell suspension (1 × 10^3^ cells/mL in RPMI-1640 medium) were added to each well, resulting in a final volume of 200 µL and final essential oil concentrations of 3.0, 2.0, 1.0, 0.5, 0.25, 0.125, 0.06, and 0 mg/mL.

The plates were incubated at 37 °C for 48 h. After incubation, 50 µL of 0.08% triphenyl tetrazolium chloride (TTC) was added to each well, and the plates were incubated for an additional 4 h. The minimum inhibitory concentration (MIC) was determined as the lowest concentration of the essential oil that caused a critical reduction in the purple precipitate (metabolized TTC), indicating the inhibition of *C. albicans* growth compared with that in the control wells.

In wells where complete inhibition of growth was observed, a sample was taken and plated onto Mueller-Hinton agar (2% glucose and 0.5 μg/mL methylene blue dye) to verify the absence or near absence of growth. The lowest concentration that resulted in no visible growth upon subculturing was determined as the minimum fungicidal concentration (MFC).

### 4.5. Time-Killing Fungal Kinetic Assay

The time–kill kinetics assay was performed using *Candida albicans* cultures adjusted to an initial inoculum of approximately 1–1.5 × 10^3^ CFU/mL in RPMI-1640 medium. The essential oil of *L. graveolens* was first dissolved in dimethyl sulfoxide (DMSO) before being added to the RPMI-1640 broth medium, at a final concentration of 0.2% (*v*/*v*). A DMSO tolerance curve previously performed demonstrated that concentrations up to 10% (*v*/*v*) did not affect the growth of any *Candida* yeast strains, confirming that the concentration used in this study was safe for antifungal testing. Nevertheless, all experiments included a DMSO control (yeast + DMSO) to corroborate the safety and reliability of the assay. The assay was carried out in sterile tubes containing 10 mL of the inoculated medium and *L. graveolens* essential oil at final concentrations of 6, 4, 2, 1, 0.5, 0.25, 0.125, and 0 mg/mL. The tubes were incubated at 36 °C with constant agitation. At predetermined time intervals (0, 3, 6, 24, 27, 30, and 48 h), 50 µL aliquots were withdrawn from each tube and serially diluted in 0.85% sodium chloride solution to obtain undiluted, 1:100, and 1:10,000 dilutions. The aliquots were plated onto Mueller–Hinton agar supplemented with 2% glucose and 0.5 µg/mL methylene blue, using three-section Petri dishes to represent the different dilutions. After incubation at 36 °C, colonies were counted to determine the number of surviving cells at each time point. All experiments were performed in triplicate to ensure reproducibility and reliability [57].

### 4.6. Germ Tube Formation Inhibition Assay

The inhibition of germ tube formation by *L. graveolens* essential oil was evaluated using a standardized protocol with *C. albicans* ATCC 10231/CDBB-L-1003 as described previously [58]. The yeast strain was initially cultured in RPMI-1640 liquid medium and incubated at 37 °C for 24 h. After incubation, the yeast suspension was then prepared in Eppendorf tubes containing 500 µL of heat-inactivated and filtered fetal bovine serum (FBS). The FBS was inactivated by heating at 56 °C for 30 min in a water bath and filtered through a 0.22 µm filter unit (Millipore, Darmstadt, Germany). A total of 10 µL of the yeast culture was added to the FBS, and the cell density of the suspension was determined using a Neubauer chamber. The initial cell density for all the samples was approximately 3.5 × 10^5^ CFU/mL. *L. graveolens* essential oil at concentrations of 2, 1, 0.5, 0.25 and 0.125 mg/mL was then added to the yeast suspensions. An untreated control was included to observe natural germ tube formation. The suspensions were mixed thoroughly by vortexing and incubated at 37 °C for 4–6 h.

Following the incubation period, the samples were observed under a microscope to assess germ tube formation. Germ tube formation was confirmed when the tubular structures showed no constriction and were 2–3 times longer than the length of the yeast cells. The number of cells that formed germ tubes was counted using a Neubauer chamber. The results were compared with those of the control to evaluate the inhibitory effect of *L. graveolens* essential oil on germ tube formation.

### 4.7. Cell Wall Integrity Assay

*C. albicans* ATCC 10231/CDBB-L-1003 was grown in RPMI-1640 liquid medium at 37 °C for 24 h. The cell suspension was prepared by diluting the culture to a concentration of 1 × 10^6^ CFU/mL in sterile saline solution. This suspension was then incubated with various concentrations of *L. graveolens* essential oil (2, 1, 0.5, 0.25, 0.125, and 0 mg/mL) for 4–6 h at 37 °C.

To evaluate cell wall integrity, the cells were stained with calcofluor-white (CFW) and propidium iodide (PI). Calcofluor-white binds to chitin and cellulose in the fungal cell wall, emitting blue fluorescence under UV light, whereas propidium iodide is a membrane-impermeable dye that penetrates cells with compromised membranes, binding to nucleic acids and emitting red fluorescence.

After incubation, 10 µL of the stained cell suspension was placed on a microscope slide, and a cover slip was applied. The samples were examined under an inverted microscope equipped with a fluorescence lamp. Calcofluor-white was observed at an excitation wavelength of 450 nm, and propidium iodide was observed at 580 nm. Images were captured to assess the structural integrity of the cell walls and the permeability of the cell membranes.

Cells with intact cell walls fluoresced blue, whereas cells with compromised cell walls and membranes exhibited both blue and red fluorescence. The presence of red fluorescence indicated cell membrane damage, allowing propidium iodide to bind to nucleic acids.

### 4.8. Biofilm Formation Inhibition Assay

The inhibitory effect of *L. graveolens* essential oil on biofilm formation by *C. albicans* was assessed using a previously described protocol with slight modifications [58,59].

An inoculum of *C. albicans* ATCC 10231/CDBB-L-1003 cells was initially cultured overnight in RPMI-1640 medium buffered with MOPS (pH 7.0) at 37 °C. Following incubation, the cells were counted in a Neubauer chamber, centrifuged at 1200 rpm for 10 min, and resuspended in fresh RPMI-1640 medium to achieve a cellular density of 1 × 10^7^ CFU/mL.

To evaluate biofilm formation, 100 µL of the cell suspension was added to each well of a 96-well microtiter plate. *L. graveolens* essential oil (1 mg/mL) solution was prepared in RPMI-1640 medium. Subsequently, 100 µL of the essential oil preparation was added to the wells containing the cell suspension, resulting in a final volume of 200 µL per well. Control wells containing only RPMI-1640 medium and the cell suspension were included to observe natural biofilm formation.

The plates were incubated at 37 °C for 24 h to allow biofilm formation. After the incubation period, the medium was carefully aspirated, and the wells were washed twice with 200 µL of PBS to remove nonadherent cells. The remaining adherent cells were air-dried for 45 min and stained with 150 μL of 1% crystal violet for 45 min. To destain the biofilms, 200 µL of methanol was added, and the plate was incubated for 15 min. From each well, 100 μL was transferred to a new microtiter plate. Optical density (OD) values were determined at 595 nm using a microplate reader (MultiskanSkyHigh Spectrophotometer, Thermo Fisher Scientific Inc., Singapore). The reduction in biofilm formation in the presence of the essential oil was determined by comparing the absorbance values of treated wells to those of untreated/control wells. The experiments were performed in triplicate to ensure reproducibility and reliability.

## Figures and Tables

**Table 1:** Chemical composition of *L. graveolens* essential oil identified by GC-MS. The table presents the compounds identified as part of *L. graveolens* essential oil, including retention times, similarity index, compound identities and Kovats Retention Index estimated values. KRIe: Kovats Retention Index estimated (column used: 30 m × 0.25 mm × 0.25 m; dead time 2.0 min). Compounds marked with an asterisk (*) are putative identifications due to their low similarity index.

Retention Time (min)	Compound	Abundance (%)	Similarity Index	KRIe
4.632	α-Thujene	3.495	91	1087.6
4.7442	1R-α-Pinene	4.657	96	1102.1
4.9446	Camphene	1.618	97	1128.2
5.2572	β-Myrcene	6.356	87	1168.6
5.5217	γ-Terpinene	4.796	97	1202.9
5.6018	α-Terpinene	11.215	90	1213.3
5.682	O-Cymene	2.643	96	1223.6
5.7461	Eucalyptol	2.494	97	1231.9
5.8102	Ocimene	0.362	87	1240.2
5.8343	p-Mentha-1,4-dien-7-ol	10.896	97	1243.3
5.9946	Terpinolene	0.646	97	1264.2
6.3873	Linalool	1.283	93	1315.0
6.4755	3-Carene *	1.176	64	1326.5
6.7	(+)-4-Carene *	0.347	62	1355.5
6.9244	(−)-Camphor	0.876	97	1384.6
7.1247	Borneol	0.685	91	1410.6
7.1568	(−)-4-Terpineol	3.121	94	1414.7
7.3572	Terpineol	0.536	95	1440.7
7.4534	Isothymol methyl ether	5.766	91	1453.1
7.5496	Thymol methyl ether	0.9	93	1465.7
7.9744	Thymol	6.894	94	1520.6
8.0625	Carvacrol	18.745	90	1532.1
8.4072	4-Hydroxy-3-methylacetophenone	0.566	94	1576.7
9.0164	Caryophyllene	3.632	99	1655.6
9.2648	α-Caryophyllene	2.301	97	1687.8
9.4171	Acetophenone *	0.834	81	1707.5
10.1305	Caryophyllene oxide	1.672	91	1800.0
	Total	98.512		

**Table 2:** Antifungal activity of *L. graveolens* essential oil against *Candida* pathogen strains. Growth inhibition halos (mm) were determined using the disk diffusion method. Minimum inhibitory concentration (MIC) and minimum fungicidal concentration (MFC) values were established through broth microdilution assays. Nystatin was used as a negative control. All results represent the means ± standard deviations of three independent replicates, ensuring the robustness and reproducibility of the findings.

Strain	Inhibition Halos (mm)		MIC (mg/mL)	MFC (mg/mL)
	Nystatin	*L. graveolens* Essential Oil		
*C. albicans* (resistant clinical isolate)	11.5 ± 0.2	30 ± 0.0	2.0	3.0
*C. albicans* ATCC 10231/CDBB-L-1003	12.0 ± 0.1	30 ± 0.0	2.0	3.0
*C. glabrata* (resistant clinical isolate)	11.4 ± 0.1	30 ± 0.0	2.0	3.0
*C. glabrata* CDBB-L-1536	11.5 ± 0.2	30 ± 0.0	2.0	3.0
*C. tropicalis* (resistant clinical isolate)	11.5 ± 0.1	30 ± 0.0	2.0	3.0
*C. tropicalis* CDBB-L-1098	11.5 ± 0.1	30 ± 0.0	2.0	3.0

## Data Availability

The original contributions presented in this study are included in the article. Further inquiries can be directed to the corresponding authors.
